# The link between number and action in human infants

**DOI:** 10.1038/s41598-022-07389-9

**Published:** 2022-03-01

**Authors:** Gisella Decarli, Ludovica Veggiotti, Maria Dolores de Hevia

**Affiliations:** 1 Université de Paris, INCC UMR 8002, CNRS, 75006 Paris, France; 2 Integrative Neuroscience and Cognition Center-CNRS UMR 8002, CNRS, Université de Paris, 45 Rue des Saints Pères, 75270 Paris Cedex 06, France

**Keywords:** Cognitive neuroscience, Neuroscience, Psychology

## Abstract

Humans' inborn ability to represent and manipulate numerical quantities is supported by the parietal cortex, which is also involved in a variety of spatial and motor abilities. While the behavioral links between numerical and spatial information have been extensively studied, little is known about the connection between number and action. Some studies in adults have shown a series of interference effects when simultaneously processing numerical and action information. We investigated the origins of this link by testing forty infants (7- to 9-month-old) in one of two experimental conditions: one group was habituated to congruent number-hand pairings, where the larger the number, the more open the hand-shape associated; the second group was habituated to incongruent number-hand pairings, where the larger the number, the more close the hand-shape associated. In test trials, both groups of infants were presented with congruent and incongruent pairings. We found that only infants habituated to congruency showed a significantly higher looking time to the test trial depicting incongruent pairings. These findings show for the first time that infants spontaneously associate magnitude-related changes across the dimensions of number and action-related information, thus offering support to the existence of an early, preverbal number-action link in the human mind.

## Introduction

Humans and non-human species share the ability to approximately extract and compare the numerosity of sets. This ability, known as the ‘number sense’^1^, is supported by the Approximate Number System (ANS) and is already functional at birth^2^. The accuracy with which the numerosity of two sets of items can be discriminated depends on their ratio: at birth newborns can discriminate two sets only if they differ for a minimal ratio of 1:3 ratio (i.e., 6 vs. 18 elements), at 6 months it follows a 1:2 ratio and at 9 months a 2:3 ratio^3,4^. The numerical acuity continues to develop into early adulthood, when it can reach a 9:10 ratio^5,6^. More recently, a study found that 4-month-olds’ acuity can be improved (from 1:4 ratio to 1:3 ratio) when infants are exposed to both visual and auditory numerosities, demonstrating that the individual numerical acuity is malleable and can be influenced by intersensory stimulation^7^.

The neural basis of the numerical processing has been found in the parietal cortex across development (in particular, the human intraparietal sulcus, IPS^8^ for a review). Activations in IPS during non-symbolic numerical tasks have been shown in 4-year-old children^9^ as well as in preverbal infants^10–12^. Interestingly, the IPS is recruited in many functions beyond the domain of numerical cognition: for instance, spatial and action-related aspects of perception, multisensory, and sensory-motor integration^13–15^. Indeed, the behavioral link between number and space is well-established in the literature. Numerical representations have been shown to be connected to spatial representations in the form of two main number-space mappings^16^: on the one hand, numbers are linked to corresponding spatial extents, and on the other hand, numbers are associated to lateralized spatial positions. Both types of mappings are fundamental and unlearned, as they have been described across the lifespan in adults^17,18^, children^19,20^, infants^21–23^, and even in newborns^24–26^.

Despite the wealth of studies on the origins and functional properties of the number-spatial link^27,28^, the association between numbers and action (hereafter we refer specifically to the magnitude of a hand opening) has received relatively little and recent attention, with some exceptions. The relevance of the number-action link has been highlighted in the ATOM view^15,29,30^, according to which the quantitative dimensions of space, time and number (and possibly others) are connected by a common metric for purposes of efficient planning and action execution. Consider, for instance, the mental computations we need to perform when we grasp one or more object/s, as we need to calculate the magnitude of the hand’s opening, as well as the distance between us and the object/s, in order to accurately perform the action.

In support of this view, studies on human adults have provided evidence of a bidirectional interference between symbolic numbers (Arabic digits) and finger/hand movements^31–36^. This interference, for example, was assessed in a study where participants were presented with Arabic digits ranging from 0 to 9 and were asked to perform a grip closure/opening based on the parity of the digit^31^. The authors identified the movement onset by assessing the first electromyographic activity; participants showed faster RTs when the closure movements were required after the presentation of small digits and, vice versa, when opening movements were required after large digits were presented^31^. In line with this study, similar findings were provided in goal-directed grasping actions^34^. Moreover, numbers’ presentation was found to influence motor judgements: the grasping action for a rod was overestimated when preceded by a small number and it was underestimated when preceded by a large number^37^.

The interference between numbers and action processing has been also demonstrated in the reverse direction. Badets and Pesenti assessed both processes in a series of experiments. One group of participants were shown one odd and one even Arabic digit (either large or small), followed by a finger movement (either opening or closure), and were asked to remember one of the two digits based on the type of movement. Another group of participants were shown either opening or closing movements, which were followed by the presentation of the pairs of digits. The authors found that participants responded more slowly in the case of large numbers when perceiving a closing movement (notice, however, that the same trend was not found for small numbers^38^). In another study, observing a closing or opening grip influenced the participants’ responses in a number generation task, i.e., participants named more often small numbers after observing a closing grip^39^. Yet, more evidence for the number-action link can be found in studies involving clinical patients. Patients with neglect, in addition to their visuo-spatial difficulties, can present a deficit also in mental number line representation^40^, and patients with Gerstmann syndrome present both numerical, spatial, and motor-related difficulties, thus presenting acalculia, agraphia and finger agnosia^41,42^.

All these studies support a link between numerical and action processing, at the behavioral level. However, the observation of this association finds its roots also at the neural level. As already mentioned, the parietal cortex is crucially involved in numerical perception as well as in goal-directed movements^14,43–45^. Specifically, grasping movements showed an activation in the anterior intraparietal cortex^13^, and monkeys showed number-selective activity in the parietal area when performing specific movements^45^, areas critically involved in numerical processing as well^46^. Andres et al., using functional magnetic resonance imaging (fMRI), assessed adult participants’ finger discrimination as well as arithmetic abilities, and provided evidence for an overlap of activity in the intraparietal sulcus (IPS) and the superior parietal lobule (SPL) bilaterally for both mental arithmetic and finger discrimination^47^. Although these studies strongly support the idea that numerosities and action are connected in the adult brain, little is known about its origins and developmental course. Intuitively, they could be associated early in life: for instance, if we think at the counting process, children systematically relate their fingers and the corresponding numerosities when learning to count, and this activity extends well into older childhood and even beyond adulthood^48–50^. Older children use fingers to express numerical concepts and to associate the “number words” with its numerical representation^51^. Moreover, this association has been highlighted also by one study demonstrating the separation between ventral and dorsal stream in young children^52^. Using principal component analysis (PCA), these authors showed that fingers gnosis, visuo-spatial abilities, non-symbolic numerical comparison tapped on the dorsal stream, while object and face recognition tapped on the ventral stream.

What are the origins of the association between numbers and action? Is it already present in development before the acquisition of counting and of formal symbols? Because adjacent areas in the parietal cortex host both numerical processing and visuo-motor abilities from very early in life^11,12,53^, it is reasonable to predict that the number-action coupling might emerge very early in development, during the first year of life. In fact, preverbal infants have been shown to take into account the information of another quantitative dimension, the size of an object, when performing actions such as grasping and reaching for an object. In particular, a large literature supports the influence of objects’ size in programming infants’ manual actions. For example, we know from previous reports that reaching activity appears in the first months of life (around 4 months^54^), that infants adapt their movements when different object sizes are presented^55^, and that their preferences are guided by the expertise they gain during development^56^. In this vein, infants as young as 6 months are able to extract the size of an occluded object by the hand grasping opening^57^ and to anticipate a goal-directed action based on the different openings previously shown to them^58^.

When considering the domain of action as a general one that recruits a variety of processes, including visuo-spatial ones, the number-space mapping previously disclosed in infants (e.g.^23^) could be considered an instance of the number-action mapping. Past research on the link between number and visuo-spatial processes has focused on infants’ performance in tasks involving either visual images that varied only in a spatial variable, such as the size of an object^23,59^, or in tasks where infants were tested in their numerical abilities while manipulating the spatial arrangement of the stimuli (e.g., left-to-right, instead of centered, presentation of the stimuli^22^). With this study we wanted to depart from this type of visuo-spatial stimulation and bring the concept of magnitude of an action into the task, so we used hand stimuli which were widely used in the adult literature to explore the link between number and action.

We created a series of images of static hands that differed in the size of the aperture in order to elicit a continuous gradient of magnitude in the action of opening the hand. Since it is well known that infants’ understanding of actions is related to their own motor abilities (e.g.^56,60^) we selected a simple movement of opening a hand. Indeed, this action can be performed and adapted by different object properties by infants as young as 4 months^54,61^. Note that although the hand shapes could activate the concept of grasping, no actual action was presented to infants. Moreover, we wanted to be consistent with the adult literature on number-action mappings (see e.g.^38^).

**Figure 1:**
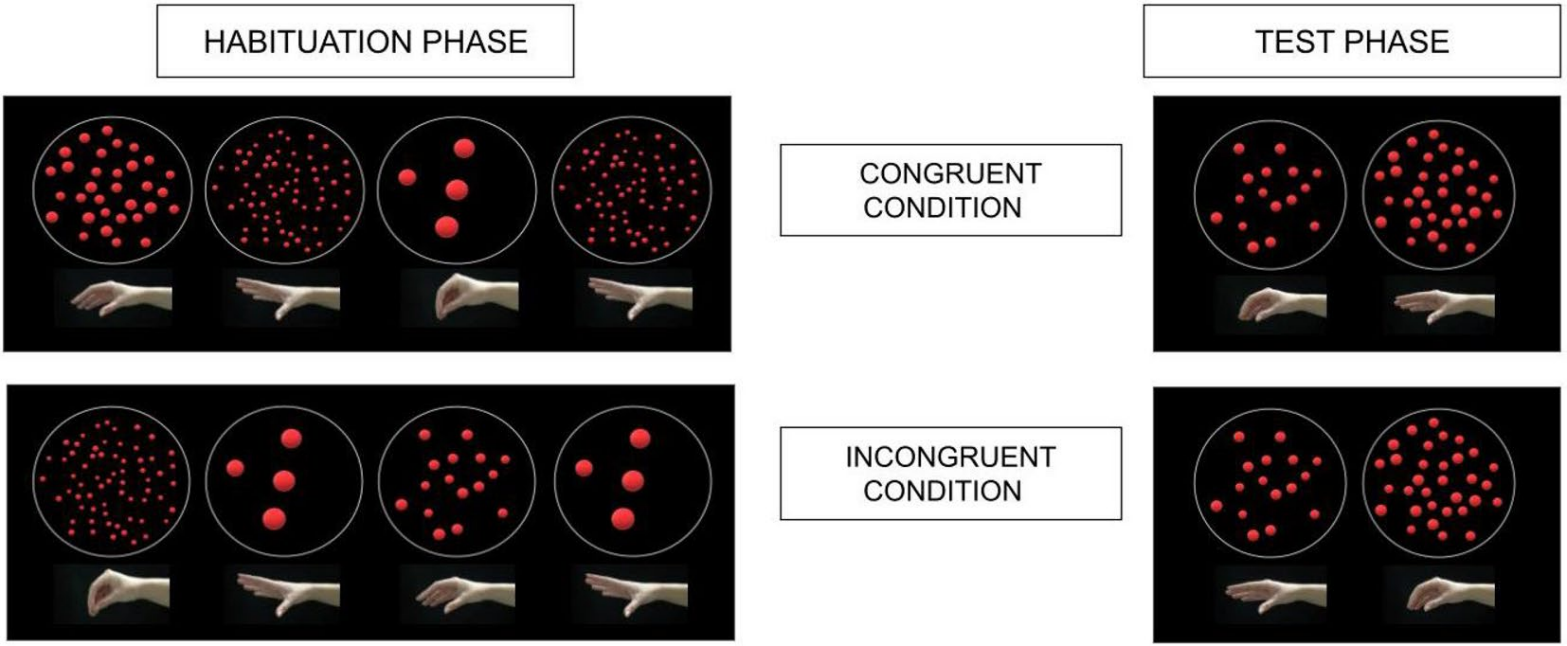
Schema of the habituation and test phases. In the habituation phase infants observed either congruent number-hand pairings or incongruent number-hand pairings. At test, all infants observed an instance of both a congruent and an incongruent pairing using new numerosities and hand openings, with values that were intermediate to those observed during habituation.

We modeled our methods on previous studies investigating the number-spatial link in infancy^23^, and tested infants’ ability to learn and generalize a rule presenting number-hand pairings using the habituation technique. In this paradigm, infants are subjected to a two-phase method: first, during the habituation phase, infants are shown multiple examples exhibiting a particular piece of information that we want them to get used to or learn. In particular, we presented infants with several trials containing a pairing between a numerosity and the magnitude of a hand shape. This phase is infant-controlled, which means that it is the infant who determines for the most part the length of this phase (i.e., single trials will continue until the infant looks away for 2 consecutive seconds, and there is a particular criterion to decide when the infant has ‘reached’ habituation; see the “Methods” section). The numerosities and hands’ openings could be either congruent (i.e., the larger the number, the larger the hand opening, so that numerosities 4, 16, 64 are paired with magnitude-corresponding hand openings, respectively, small, medium, large) or incongruent (i.e., the larger the number, the smaller the hand opening, so that numerosities 4, 16, 64 are paired with inversely corresponding hand openings, respectively, large, medium, small; see Fig. 1). Half of the infants are habituated with congruent pairings while the other half with the incongruent ones. In the test phase, which starts as soon as the infant has been habituated, infants are shown new information (new numbers—8 and 32—and new hand openings—small-medium and medium-large) that could be considered familiar (when the new information conforms at an abstract level to the information shown during habituation) or novel (when the new information contrasts with the information shown during familiarization). If infants are sensitive to the number-action mapping and able to extract the relevant information during habituation (in this case the rule relating numbers and hand shapes) then they should look longer (recover attention) at the novel information presented at test. In particular, if infants are sensitive to the number-action association and able to generalize the rule, when they are habituated to congruency, they should look longer at the (novel) incongruent numerosities-hand opening. Inversely, if the number-action mapping obeys to a specific (congruent) direction, and therefore does not emerge with any arbitrary type of pairing, then infants habituated to incongruent number-action pairings should not be able to learn the rule during habituation and therefore not generalizing it at test, exhibiting similar looking times to both congruent and incongruent test trials.

## Results

**Figure 2:**
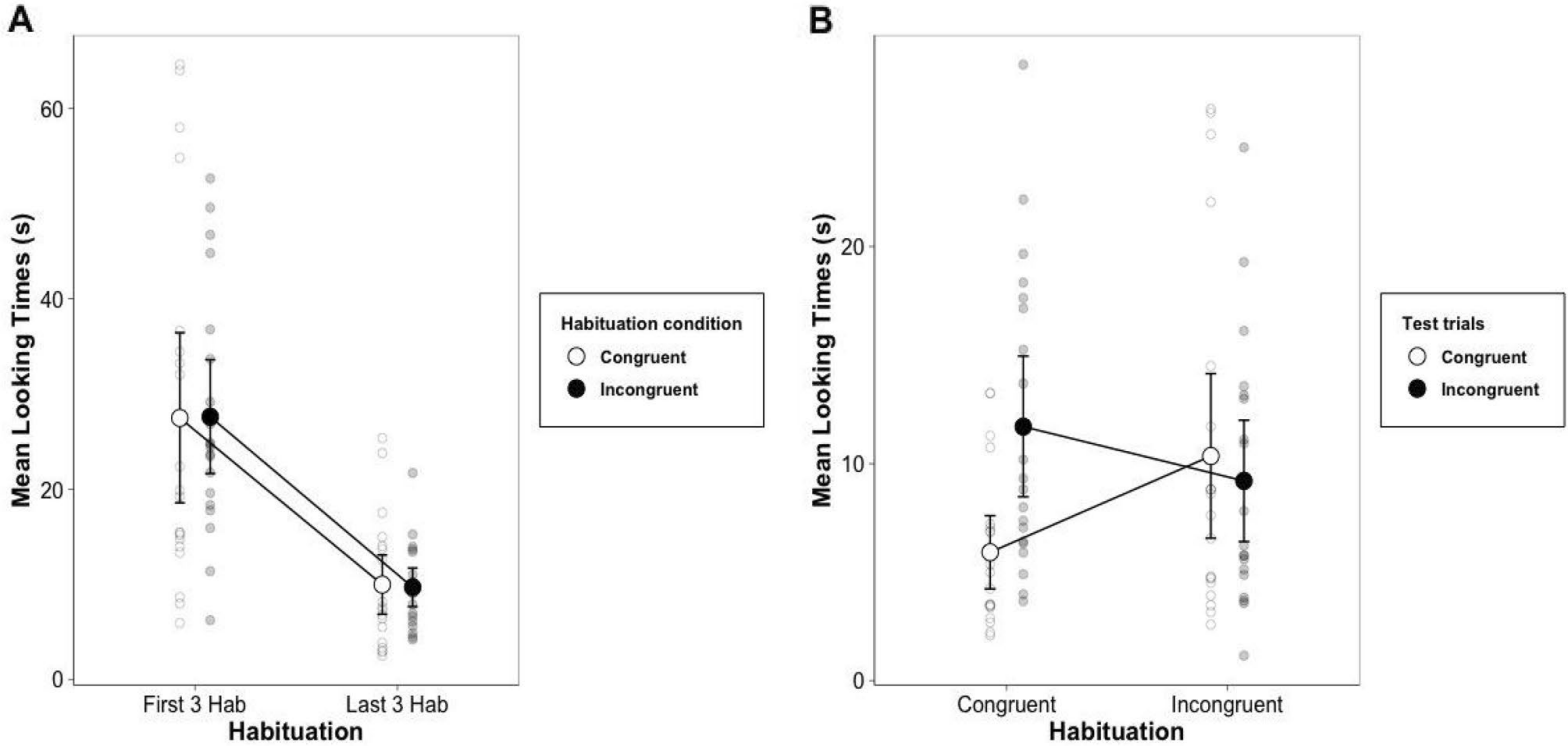
(**A**) Mean looking times in the congruent and incongruent habituation conditions for the first and last three trials of habituation, showing no differences across the two habituation conditions. (**B**) Mean looking times in the first and last three habituation trials for the two test-trials, showing a significant looking time difference between the two test-trials for infants habituated to the congruent condition, and no difference for infants habituated to the incongruent condition. Error bars represent 95% confidence intervals.

Infants observed an average of 8.1 habituation trials. No differences were found in the number of habituation trials observed by infants in the congruent and incongruent conditions (congruent habituation: *M* = 8.85; incongruent habituation: *M* = 7.35; *p* = 0.08; BF_10_ = 1.06, anecdotal evidence). Infants showed habituation from the first 3 (*M* = 27.53 s, *SD* = 16.01 s) to the last 3 trials (*M* = 9.81 s, *SD* = 5.55 s), *t*(39) = 8.76, *p* < 0.001, BF_10_ > 100, extreme evidence). Moreover, we did not find significant differences when infants were habituated to the incongruent pairings compared to the congruent ones either in the first 3 trials (habituation to congruent pairings: *M* = 27.47 s, *SD* = 19.07 s; habituation to incongruent pairings: *M* = 27.59 s, *SD* = 12.75 s; *t*(38) = − 0.02, *p* = 0.98, *d* = 0.007, BF_10_ = 0.31, anecdotal evidence, see Fig. 2A) or in the last 3 trials (habituation to congruent pairings: *M* = 9.95, *SD* = 6.66; habituation to incongruent pairings: *M* = 9.67 s, *SD* = 4.33 s; *t*(38) = 0.16, *p* = 0.87, *d* = 0.05, BF_10_ = 0.31, anecdotal evidence).

Infants’ looking times at test were analyzed using an analysis of variance (ANOVA) with habituation condition and first-test order as between-subject factors, and test-trial type as within-subject factor (for all the factors: congruent vs. incongruent). We found a significant habituation condition × test-trial type interaction (*F*(1,36) = 7.1, *p* = 0.01, η^2^_G_ = 0.08, see Fig. 2B). When infants were habituated with the congruent condition, they looked longer at the incongruent test pairings (*M* = 11.71 s, *SD* = 6.91 s) compared to the congruent ones (*M* = 5.91 s, *SD* = 3.61 s; *t*(19) = − 4.01, *p* = 0.001, *d* = 0.89; BF_10_ = 47.17; very strong evidence). Moreover, 16 out of 20 infants looked longer at the incongruent test trial (binomial test: *p* = 0.01).

In contrast, infants looked comparably long at the two test trials when they were habituated to the incongruent pairings (congruent test-trial: *M* = 10.35 s, *SD* = 8.09 s; incongruent test-trial: *M* = 9.21 s, *SD* = 5.98 s; *t*(19) = 0.54, *p* = 0.59, *d* = 0.12; BF_10_ = 0.26; anecdotal evidence). Thirteen out of 20 infants looked longer at the incongruent test-trial (binomial test: *p* = 0.26). We did not find other effects or interactions (all *p*s > 0.07).

## Discussion

Despite the recent interest on the mutual influence between action and numbers^31,32,35,62^, to date no studies have investigated the origins of this link in humans, specifically in the first year of life. Therefore, this study was designed to test whether 7- to 9-month old infants are able to spontaneously extract, and generalize, the congruency between couplings showing action and numerosity information. We presented streams of images with different sets of dots that were accompanied by different hands’ openings. The pairings could be either congruent (i.e., the larger the number, the larger the hand opening), or incongruent (i.e., the smaller the number, the larger the hand opening). We measured infants’ looking times during test trials containing new examples of both congruent and incongruent number-action pairings. We found that infants habituated to a congruent number-hand pairing looked longer at new, incongruent pairings at test, while infants habituated to an incongruent pairing did not show any differential looking times at test. These findings suggest that infants were able to extract and generalize the number-action rule when habituated to stimuli showing a systematic congruency of magnitude changes across the dimensions of action and number. In contrast, when receiving an incongruent pairing, infants failed at extracting and/or generalizing this rule at test. The magnitude dimensions of action and number might therefore be connected in a unidirectional, only congruent way, supporting the view that these two sources of information are linked early in life in the human mind.

Although our hand images were motionless and therefore strictly no information of active movement was presented, the hand shapes could have evoked the action of grasping. As a result, infants might have been sensitive to the individual size of the single elements in the numerical arrays when interpreting the appropriate hand shapes. However, as in previous research using similar methods^23^, the numerical displays were designed such that the larger the number, the smaller the individual size of the single elements (i.e., an inverse relationship between number and individual size), a manipulation routinely done to control for the overall area of the numerical displays. In this way, infants could have established a relationship of congruency between the individual size of the dots and the magnitude of the grip aperture, by relating the smallest quantity to the largest hand opening; however, the findings reveal the exact opposite finding. Moreover, and critically, during test trials the size of the individual objects was constant across the two numerosities, so that this cue could not help infants to solve the task. Finally, we would like to point out that infants, when encountering arrays of large numerosities, do not pay attention to the individual's properties, but create a representation of the approximate numerosity of the set^63,64^. In our study, we were careful to employ only numerosities that pertain to the large range (i.e., 4/16/64 dots in the habituation trials and 8/32 dots in the test trials). The findings of our study are therefore consistent with the idea that infants created a representation of numerosity and that they established a relationship between this numerosity representation and the magnitude of hand aperture in a consistent way, but failed to learn the relationship when they were related in an inconsistent way. Moreover, our results are in line both with previous findings of infants at the same age establishing a mapping between numerosity and length^23^, as well as with studies showing that when large arrays are presented infants create a representation of the numerosity of the whole set^63,64^.

Secondly, these findings are in line with ATOM’s view^15,29,30^. Indeed, they support the idea that at a certain level of abstraction there is a common magnitude code shared between a variety of quantitative dimensions, including the magnitude of an action, which is already present in the first steps of development. In this vein, hand movements and numerosity perception abilities can be seen as being both supported by the parietal lobes, in line with children’s studies describing the functional properties of the ventral and dorsal streams (one supporting face/object identification and the other supporting math and fingers processing; see^52^). Notably, some studies found this dissociation already in the infant brain^12,53^.

On the other hand, while our results shed some light on the origins of the number-action link, by showing its presence in the first months of life, they open the question of the functionality and developmental course of this association. When is the association between numbers and action functional? Is the structure of the brain early in life already prepared to integrate this kind of information? One might speculate that numbers and actions become linked only during development, thanks to the continuous learning of the infant in interaction with the surrounding environment. In this view, experience plays an important role in modelling the association between them. Starting from the first months of life infants learn a lot about the world surrounding them: reaching and grasping are the most powerful tools that infants have to explore the objects and soon they are able to adjust a movement on the basis of the objects’ features^61,65^. In this sense, in our experiment, looking at hands’ opening shapes might have evoked a grasping action that might in turn have been calibrated on the basis of the numerosity shown. This calibration could be learnt only thanks to the interaction with the environment.

However, another possibility is that at birth the information of number and action understanding is already functional and interact with each other in the brain. In fact, as for other mappings between magnitude dimensions, such as number–space–time, the number-action link might be present at the start of postnatal life. Humans start to perform actions from the first hours of life and need to integrate multiple sources of information to finalize them. Thus, newborns might possess a rudimentary structure that allows them to associate numerosities and actions in a coarse and imprecise way. During development, and possibly through experience/maturation, this structure might gain in complexity. This idea is in line with previous findings that highlighted the integration of different domains of perception (i.e., numbers and space) at birth^24,25^. Moreover, the use of specific actions plays a pivotal role in the acquisition of numerical abilities, and certain actions, such as pointing and grasping, continue to exert an influence in numerical processing during the adult years^62^. In fact, toddlers start very early to use their fingers to help them in the counting process and in associating each counted object to its numerical symbol. Through the performance of actions, such as pointing and finger-counting, children make their way into understanding the principle of cardinality and the one-to-one correspondence^48,50^. Finally, our results clearly highlight that the number-action link is already present prior to formal schooling and might form the basis for exploiting this association later in life in the acquisition of more sophisticated abilities. Thus, an important future direction could be to investigate the developmental trajectories of the numbers-action link, including its assessment at birth.

In conclusion, we provide the first evidence for an association between numerosities and depicted actions in the first year of life. This finding opens the possibility that not only different magnitudes are strictly connected early in life, but they are simultaneously involved in action processing already in infancy.

## Methods

### Participants

Participants were forty full-term infants (18 female and 22 male; mean age = 8 months and 10 days, SD = 0.49; months; age range = 7 months and 13 days–9 months and 5 days). Nine additional infants were excluded due to fussiness/cry/lack of interest (N = 7), parental interference (N = 1) or test-trial looking times more than 3 standard deviations from the overall group mean (N = 1). The procedure was approved by the Université de Paris Ethics Committee and met the requirements of the Declaration of Helsinki.

### Materials

Stimuli were composed of images containing dots of different numerosities and different hands’ openings. The numerosities consisted of 4, 8, 16, 32 and 64 dots. Both the size of the items and of the array in the stimuli were controlled: overall area was controlled during habituation and item size was inversely related to number; during test trials, item size was constant and overall area was positively correlated with number. The size of the dots could vary between 0.30 and 1.69 cm (4 dots: 1.18–1.69 cm; 8 dots: 0.84–1.20 cm; 16 dots: 0.59–0.84 cm; 32 dots: 0.42–0.59 cm; 64 dots: 0.3–0.42 cm). For arrays of 4 dots circle diameters could vary between 1.36° and 1.94° visual angle, 8 dots between 0.96° and 1.38°, 16 dots between 0.68° and 0.97°, 32 dots between 0.48° and 0.68°, and 64 dots between 0.34° and 0.48° visual angle (50 cm from the monitor).

The hand images consisted of five different openings and angles of a right hand (see Fig. 1): the distance between thumb and index of the hands could be 0 cm, 2 cm, 5 cm, 8 cm, and 10 cm. During the habituation trials infants were shown the images with 0 cm, 5 cm, and 10 cm of hand opening paired with 4 dots, 16 dots and 64 dots respectively (for the congruent habituation condition), and with 64 dots, 16 dots and 4 dots respectively (for the incongruent habituation condition). During the test trials infants were shown the images with 2 cm and 8 cm of hand opening paired with, respectively, 8 dots and 32 dots (for the congruent test trial), and to, respectively, 32 dots and 8 dots (for the incongruent test trial).

Each trial consisted of the presentation of an array of dots centered in the upper part of the screen for 1000 ms. Afterwards, with the dots array still visible, the hand appeared centered in the lower part of the screen, and both images remained visible together for 1000 ms. Each number-hand pairing was separated by a black screen lasting 500 ms. Following the methods in^23^, the order of the number-hand pairings was pseudorandom, so that no immediate repetition of a number-hand pairing, nor three consecutive ordered pairings (either increasing—small, medium, large—or decreasing—large, medium, small), could be presented. The presentation of the trials took place on a computer screen (high-definition screen resolution 1920 × 1080 pixels and a 23″ display area). We used E-Prime 2.0 software to present the stimuli and to register online the infants’ looking times.

### Design

During the habituation phase half of the infants observed the congruent number-hand pairings (the larger the numerosities, the greater the hands’ opening), while the other half observed the incongruent number-hand pairings (the larger the numerosities, the smaller the hands’ opening; see Fig. 1). Participants were randomly assigned to one of these habituation conditions, congruent or incongruent. In the test phase, all infants observed a sequence of both types of pairings, congruent and incongruent, in two trials. The order of the test trials was counterbalanced across participants.

### Procedure

The study took place in a dark and silent room. Infants were seated on a parent's lap in front of a computer screen and they were surrounded by curtains to avoid distractions. Before starting the experiment, parents were instructed not to interact with the infant and not to point at the screen. A camera was located above the screen and it was connected to a monitor, from which an experimenter could observe and register online infants’ looking times. This camera also allowed us to register the infant's behavior for offline coding. Half of the participants were randomly assigned to the congruent habituation condition (e.g., large number-open hand) and the other half to the incongruent habituation condition (e.g., large number-closed hand).

Each trial was composed of a sequence of two images that appeared consecutively and that remained visible together for 1000 ms. Thus, a complete image (including the dots array together with the hand opening) remained for 1000 ms, while a single trial was constituted by a collection of complete images (e.g., small number with small opening, large number with large opening, medium number with medium opening…) that appeared until the infant looked away for 2 consecutive seconds. Test trials were constituted each by the alternation of two numerosities paired with two hand openings (in one trial the pairing is congruent, in another trial the pairing is incongruent). Also in this case, for each given trial, infants observed a stream of images containing two numerosities paired with two hand openings, and these trials could contain either congruent or incongruent pairings (half the infants were tested first with the congruent test trial). Each trial presented the numerosities 8 and 32 paired each with a hand opening; in the congruent test trial the pairing was made such that the 8 dots were paired with a small-medium hand opening, and the 32 dots with a large-medium hand opening, while for the incongruent test trial the pairing was reversed. Thus, each image presented a specific numerosity and a specific hand opening.

For both the habituation and test phases, each trial (a sequence of number-hand images) remained visible until the infant looked away for 2 s continuously (or looked for a maximum of 120 s). An 'attention-getter' appeared centered on the screen at the beginning of each trial in both the habituation and test phases, and was terminated by the experimenter as soon as the infant looked at the screen, initiating the trial. The test phase started when the infant reached the criterion of a 50% decrease in looking time over three consecutive trials compared to the looking times of the first three trials that totaled at least 12 s^23^. Thus, participants could observe a minimum of six habituation trials and a maximum of 14 habituation trials. For the test phase, regardless of the number of trials seen during habituation, all infants were presented with two trials, one congruent and one incongruent pairing sequence. The duration of the experiment could vary from 5 to 10 min.

### Offline coding and data analysis

For offline coding, each infant was analyzed by two experienced observers who coded offline the data, and an average was made between the two closest codings, including the online coding. The intercoder agreement was high (90%).

All the analyses were computed using R^66^. We reported both frequentist and Bayesian analyses and the evidence associated with Bayes factors (BFs) was defined as “anecdotal” (1/3 < BF < 3), “moderate” (BF < 1/3 or BF > 3), “strong” (BF < 1/10 or BF > 10), “very strong” (BF < 1/30 or BF > 30), or “extreme” (BF < 1/100 or BF > 100)^67^.

### Ethics approval

This study was reviewed and approved by the Ethics Committee of the University of Paris (CER U-Paris: 2021-83-DECARLI-DE HEVIA; N° IRB: 00012021-83). Written informed consent to participate in this study was provided by the participants' legal guardian. The study meets the requirements of the Declaration of Helsinki.
